# Pilot Simulation Task Trainer for Prehospital Management of Neck Hemorrhage

**DOI:** 10.5811/westjem.47912

**Published:** 2026-05-03

**Authors:** Sarah Sussman, Luigi Melaragno, Eric Nisenbaum, Megan Malara, Rachel Herster, Chipper Orban, Matthew Marquardt, Catherine Haring, Kimberly G. Harmon, Kyle VanKoevering

**Affiliations:** * The Ohio State University Wexner Medical Center, Department of Otolaryngology – Head and Neck Surgery, Columbus, Ohio; † University of Cincinnati College of Medicine, Cincinnati, Ohio; ‡ The Ohio State University Center for Design and Manufacturing Excellence, Columbus, Ohio; § The Ohio State University, College of Medicine, Columbus, Ohio; || The University of Washington, Department of Family Medicine, Seattle, Washington

## Abstract

**Introduction:**

Traumatic injuries are the leading cause of death in the U.S. of persons < 45 years of age, with 5–10% of all traumas caused by penetrating neck injuries (PNI). The neck contains several large blood vessels that supply the brain; thus, exsanguination is the leading cause of fatality in PNI. Vascular neck trauma is common in assaults, motor vehicle accidents, battlefields, and sporting events, particularly in ice hockey. There is a general lack of guidance on prehospital management of these injuries, and educating first responders, medics, and sports trainers on how to manage these complex injuries is challenging due to high costs, limited availability, and ethical considerations regarding use of cadavers or live animals. Here, we describe the development of a prehospital PNI-hemorrhage curriculum paired with a novel hands-on simulator and its pilot implementation with a group of professional hockey athletic trainers.

**Methods:**

We conducted a literature review to understand previously proposed algorithms for PNI, traumatic life support, combat trauma, and massive hemorrhage. Concepts from each of these algorithms were considered when determining the key steps of managing a PNI and how these should differ from previously proposed algorithms. We developed a synthetic medical simulator and training curriculum in conjunction with the National Hockey League (NHL) to create a training program for athletic trainers and team physicians to improve rapid response. The simulator was designed using computed tomography of a human neck and was fabricated to mimic the material properties of human tissue.

**Results:**

The algorithm for prehospital management of PNI was developed in three fundamental steps: 1) identify venous vs arterial bleeding patterns; 2) control the hemorrhage; and 3) transfer the patient to a trauma center. The synthetic medical simulator allowed for the simulation of arterial and venous bleeding and was used to train 180 NHL athletic trainers and physicians at their annual meeting in 2024. Voluntary quantitative and qualitative post-training feedback obtained from 46% of trainers who participated was very positive (overall rating 4.7/5).

**Conclusion:**

Penetrating neck injuries are high-risk events that first responders are generally undertrained to manage due to their rarity. Simulation is effective to potentially improve the outcomes of these scenarios, and the use of synthetic medical simulators is cost effective. We developed a novel algorithm, medical simulator, and training curriculum for the management of PNI in conjunction with the NHL for training athletic trainers and physicians.

## INTRODUCTION

Trauma is the leading cause of death among U.S. residents < 45 of age, according to data from the U.S. Centers for Disease Control and Prevention. Penetrating neck injury (PNI) represents 5–10% of all trauma cases and are among the most life-threatening.^1^ They are often catastrophic injuries due to exsanguination, airway compromise, aerodigestive injury, or neurological injury. Vascular neck injuries in particular have a high rate of morbidity and mortality due to the level of acuity and the time it takes to reach medical attention.^2^,^3^ The neck is a complex anatomical region containing vital structures including large vessels that supply the brain, the aerodigestive tract, and cranial nerves.^4^–^6^ This area is vulnerable to injury as it is relatively exposed and anatomically unprotected, with only the sternocleidomastoid muscle providing a barrier over the deeper vascular system. The common carotid artery is the highest pressure vessel in the neck, with a mean arterial pressure of 90–150 millimeters of mercury (mm Hg) and a flow rate of approximately 500 milliliters (mL) per minute.^7^ The deep venous system of the neck includes the internal jugular vein, which is a lower pressure system with a typical pressure of 6–8 mm Hg. However, the jugular vein can have a flow rate of up to 650 mL/minute, which lends to the potential for massive hemorrhage with compromise of the jugular vein.^8^

Vascular injury may include bleeding, occlusion, dissection, pseudoaneurysm, or arteriovenous fistula.^6^,^9^ Arterial injury occurs in approximately 25% of penetrating neck injuries with carotid artery involvement in about 80% and vertebral artery in 43% of these injuries.^10^,^11^ Mortality for all vascular injuries is 50–60%.^12^,^13^ Exsanguination is the primary cause of acute mortality in these vascular injuries and, on average, the bleed-out time varies between 2–5 minutes, as per the STOP THE BLEED campaign.^14^ Thus, in the acute, prehospital setting, prevention of exsanguination is critical for survival of these injuries.

Penetrating neck injuries can also be seen in assault, motor vehicle accidents, and sporting events. Ice hockey has had several well publicized incidents of PNI due to the presence of bladed skates. In 1989 a professional hockey goalie had his jugular vein severed by the blade of an opponent. His athletic trainer/therapist, a former U.S. Army medic, responded promptly by holding firm pressure until he was taken to the hospital.^13^,^14^ In 2008 another professional player suffered injury to his external carotid artery and lost five units of blood requiring emergent surgery.^15^ Both players eventually made a full recovery. In 2022 and 2023, two amateur hockey players exsanguinated before arriving at the hospital after suffering neck laceration during games. These incidents prompted investigation into the use of neck guards as well as training hockey players and staff in how to respond to these injuries.^16,17^

**Population Health Research Capsule**

What do we already know about this issue?

*Penetrating neck injury with involvement of the great vessels is associated with significant morbidity and mortality, yet there is very little training or guidance for pre-hospital management*.

What was the research question?

*We aimed to develop a hands-on simulator and simple algorithm to teach first responders and athletic personnel basic strategies for managing hemorrhage from penetrating neck injuries*.

What was the major finding of the study?

*The simulator, algorithm and instructional course were well received with an overall rating of 4.7/5*.

How does this improve population health?

*This training algorithm can help prepare first responders for management of catastrophic hemorrhage from penetrating neck injuries*.

Treating PNI has historically focused on the anatomical classification system described by Monson et al in 1969, which divides the neck horizontally into three superposed zones.^18^ More recent literature has shifted the focus toward “hard signs” and evolving endovascular management strategies in lieu of open exploration. The American College of Surgeons Advanced Trauma Life support (ATLS) program teaches a simple mnemonic ABCDE—Airway, Breathing, Circulation, Disability, Exposure—that is the cornerstone of trauma triage in the emergency setting. Battlefield ATLS has traditionally followed ATLS principles. However more recently, this algorithm has been amended to include <C>ABCDE for catastrophic hemorrhage’ followed by the conventional ABCDE algorithm.^19,20^ Specifically for massive hemorrhage, the American College of Surgeons produced a program in 2015 entitled “STOP THE BLEED,” which provides prehospital training that focuses on applying pressure to the source of bleeding and the use of tourniquets to decrease blood loss. The neck is unique in that a tourniquet cannot be used due to risk of airway compromise, asphyxiation, and ischemic stroke. These algorithms outline a hierarchy of initial life-saving measures that can be applied in the field to any trauma situation, civilian or combat, and emphasize the need to control catastrophic hemorrhage as the primary step.^21,22^ However, despite several well-established and logically sound trauma guidelines, airway management algorithms, and in-hospital recommendations for vascular neck injuries, there remains a gap in the development of prehospital management for vascular neck injuries.

Patients with vascular neck injuries can decompensate before reaching definitive medical care; therefore, we propose a simplified algorithm and novel simulation task trainer to teach athletic trainers or first responders prehospital management of vascular neck trauma while transporting to the nearest trauma center. To evaluate this algorithm and model, we implemented it among a group of professional hockey athletic trainers. In an athletic practice setting, even at the professional level, athletic trainers are frequently the highest level of medical care immediately available at the time of PNI. As such, they represent an ideal test case for our algorithm. We also describe the creation of a novel simulator that has been used for hands-on teaching of this algorithm to athletic trainers and team physicians in the National Hockey League (NHL).

## METHODS

**Figure 1:**
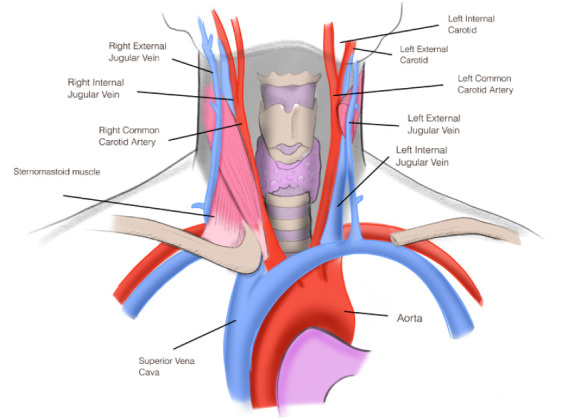
Basic anatomy overview of the human neck and major blood vessels used in a novel curriculum designed to teach prehospital management of penetrating neck injuries.

In conjunction with the NHL medical teams, we developed a curriculum around the simulation trainer and our proposed algorithm. This simplified curriculum is geared toward educating the general population and healthcare professionals, such as athletic trainers, with at least limited medical expertise for prehospital stabilization of PNI with significant hemorrhage. The curriculum focused solely on vascular injuries and did not include content related to airway management or other associated injuries. With this curriculum, participants first were given a basic anatomy overview of the human neck and major blood vessels, including key facts about blood flow, pressure, and historical outcomes of PNIs (Figure 1). This was followed by an explanation of our three-step algorithm.

**Figure 2:**
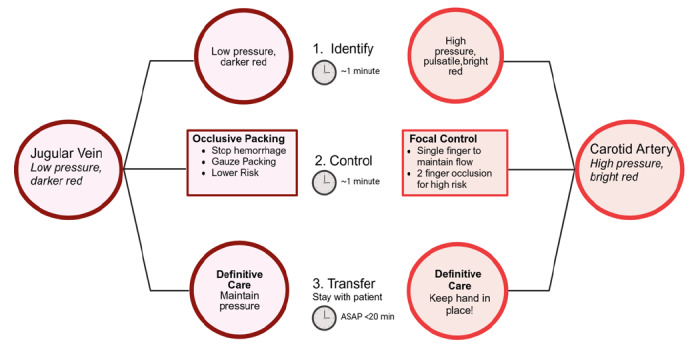
Proposed algorithm for prehospital management of acute vascular injury developed as part of a simulation-based curriculum for first responders and professional athletic trainers. *ASAP*, as soon as possible.

The algorithm was developed by synthesizing current trauma data, national and international trauma management guidelines, and experience from in-hospital management strategies aligned through consensus among the authors who are trained head and neck surgeons. The proposed prehospital treatment strategy consisted of three fundamental steps: 1) identify primarily venous vs high-volume arterial bleeding; 2) control the hemorrhage; and 3) transfer the patient to the nearest trauma center (Figure 2).

### Step 1: Identify

A systematic approach to management of vascular neck trauma is critical. First, it is important to consider the mechanism of injury and environment to ensure the safety of the patient and caregivers. Full exposure of the neck is required, and evidence supports cervical spine collar is not necessary unless there is a focal neurologic deficit or a high index of suspicion for spinal cord injury.^23^ In our algorithm, once a hemorrhagic PNI is identified, step 1 is for the emergency responder to identify whether a venous (high flow, low pressure, and non-pulsatile) and/or arterial bleed (brighter red, high pressure, and pulsatile) is present, as management strategies differ. The wound must be inspected by gently distracting the wound and observing for high-flow pulsatile bleeding.

### Step 2: Control

**Figure 3:**
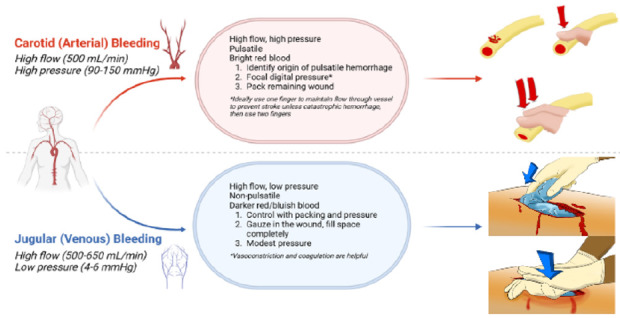
Methods for managing carotid (arterial) vs jugular (venous) bleeding in a simulation-based curriculum for prehospital responders and athletics trainers.

Once the primary source of bleeding is identified, it is critical to control the hemorrhage as quickly as possible to prevent hemorrhagic shock or exsanguination. Venous bleeding (internal or external jugular veins) can be controlled with compression achieved by packing the wound with standard gauze to fill the laceration using a finger-over-finger technique and then applying broad pressure (Figure 3).

Unlike in other areas of the body, we recommend using standard gauze to control PNI rather than hemostatic gauze, as hemostatic agents could be deposited into an injured carotid or vertebral artery and result in embolic stroke. Proper packing and broad surface pressure are highly successful in controlling superficial and/or high-volume venous bleeding as it is a low-pressure system, and vasoconstriction and coagulation will further assist. Flow in the jugular vein can typically be completely obliterated without clinical consequence.

Should a carotid or vertebral artery injury be suspected, however, broad packing techniques are much less likely to be successful in controlling a high-pressure hemorrhage. Thus, the control technique is different for a suspected large-caliber arterial hemorrhage. It is critical to identify the origin of the pulsatile hemorrhage and apply focal digital pressure directly on the vessel either at the site of vessel injury or slightly above and below the area of hemorrhage. Focal digital pressure can seal a sidewall injury, which will allow intraluminal flowthrough and brain perfusion. Alternatively, two-finger obliteration of the arterial flow can successfully control the hemorrhage, but it does increase the risk of reduced brain perfusion and stroke. Control of the hemorrhage must be prioritized over stroke risk. If the patient has sustained an arterial injury, an internal jugular vein injury is also very likely. Thus, the next step is to pack the remaining wound around the finger following the same finger-over-finger techniques illustrated above. The finger controlling arterial hemorrhage remains in the wound until definitive care is reached.

The Foley catheter balloon-tamponade technique has been described to control bleeding in difficult-to-reach areas; however in the field, a Foley catheter is rarely available and not advisable. Consensus literature strongly advises against attempting to control bleeding with hemostats, as blindly clamping the vessel is often ineffective, may cause further injury, or prevent future repairs in the hospital setting.

### Step 3: Transfer

Once bleeding is temporized, all patients with PNI or vascular injury should be transported immediately to the nearest trauma center. The primary caregiver should keep their hands in position, holding constant pressure until hospital delivery. Patients can rapidly decompensate. Monitor vital signs as able, wakefulness, and signs of stroke, which may include somnolence, blown pupil, or contralateral hemiparesis.

### Developing the Task Trainer

In addition to the above proposed algorithm, we describe a novel task trainer that was developed in conjunction with the NHL for a training curriculum for over 180 athletic trainers and team physicians. Using computed tomography of a human neck, 3D printing, and silicone molding, our team developed a synthetic model of a PNI that provides a realistic representation of vascular anatomy. The model includes an outer layer of EcoFlex 00–30 silicone (Reynolds Advanced Materials U.S., Inc, Macungie, PA) with a Shore00 durometer (PTC Instruments, Los Angeles, CA) of 30, to mimic the durometer of skin, and an inner layer of EcoFlex 00–10, which has a Shore00 Durometer of 10, similar to subcutaneous tissue. The inner “subcutaneous” layer surrounds two channels lined with rubber tubing representing the carotid artery and jugular vein. The tubing used to simulate the carotid was made of an elastic latex rubber material 10 mm in diameter with a 1.5-mm wall thickness. The tubing used to simulate the jugular was made of a biocompatible silicone rubber and was 14 mm in diameter with a 1-mm wall thickness. The increased thickness and elasticity of the carotid tubing compared to the thinner, more compliant jugular tubing mimicked the material properties of the true anatomical structures.

**Figure 4:**
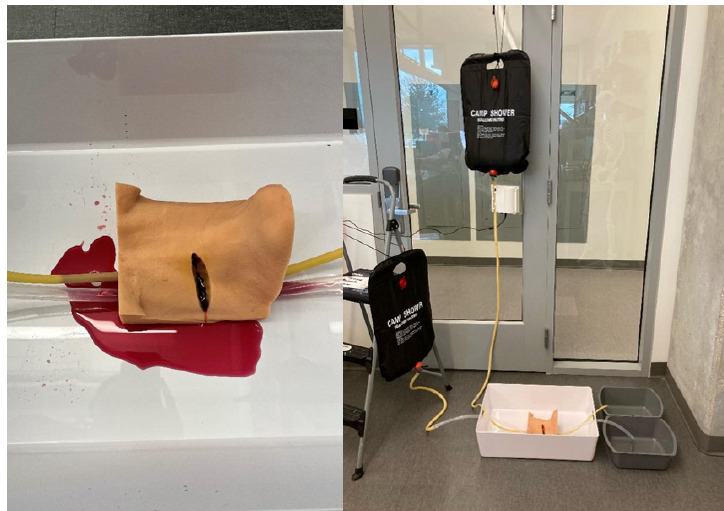
Synthetic model of a penetrating neck injury equipped with artificial, blood-like fluid (left) and a demonstration of the separate venous and arterial flow systems (right). The arterial reservoir is set higher than the venous reservoir to facilitate a greater blood pressure in the carotid artery channel and is equipped with a pump mechanism to create a pulsing flow.

The model includes a built-in laceration penetrating through both layers of silicone to the vascular channels. The vascular tubing exits the model and runs to a camp shower filled with artificial, blood-like fluid. The carotid artery system is equipped with a simple piston motor that periodically occludes the tubing, resulting in pulsative flow from and the blood reservoir (camp shower), which is set at a height of 160 cm to facilitate a pressure of about 120 millimeters of mercury (mm Hg) through the simulator. The jugular vein system reservoir was set at a height of 10 cm to facilitate a pressure of about 7 mm Hg through the simulator. The different fluid pressures in the arterial and venous systems allowed the model to realistically represent the physiologic pressures seen from jugular venous or carotid arterial hemorrhage. The simulation blood was also dyed darker red for the jugular system (Figure 4), while the arterial system was brighter red to maximize realism. In total, the materials for the simulator cost approximately $185 U.S. Synthetic simulated models are a cost-effective and reproducible method that provide recurring training opportunities, thereby improving operator response to traumatic events.

Due to the nature of the conference, no formal data could be collected; however, a post-course feedback survey was administered, and voluntary feedback related to the carotid simulation experience was reviewed.

## RESULTS

The curriculum was successfully deployed to over 180 participants including NHL team physicians and athletic trainers. Following completion of the curriculum, participants underwent simulated training sessions to practice a realistic response to a vascular neck injury “in the field.” After an initial introduction, participants were asked to respond immediately to a player down on the ice. The initial hands-on training began with active exsanguination from a simulated jugular vein laceration. During this simulation, participants gained experience exploring the wound to identify the type of bleed. They then practiced proper finger-over-finger packing techniques with conventional gauze for a large-volume venous bleed, which was uniformly successful in controlling the hemorrhage.

After the initial venous training session, a debriefing was held to review key concepts and questions. The exsanguinated blood was collected back into the venous reservoir, and a catastrophic arterial hemorrhage was then simulated. Similarly, participants were again instructed to respond immediately to a player down on the ice. They again explored the wound and were able to quickly identify the high-pressure and pulsatile bleeding of an arterial hemorrhage. They were guided to then digitally explore the wound and practice the 1- and 2-finger techniques for direct digital control of an arterial hemorrhage followed by gauze packing. During the simulation, participants could observe the difference in brain perfusion with the 1- and 2-finger techniques by observing the flow through the vessel at the distal end to simulate brain perfusion and feel the pulsatile nature of the carotid artery. A similar debrief was held at the conclusion of the simulation.

This hands-on training experience was facilitated by two otolaryngology head and neck surgeons with expertise in trauma management. Open discussion allowed for tailored teaching during each session. The material was delivered to all team trainers and team physicians at the annual NHL medical conference. At the completion of the hands-on training session, participants received a one-page summary handout to help reinforce learning principles. Due to conference requirements, formalized feedback data could not be collected; however, informal voluntary survey responses indicated that the session scored a composite 4.71 of 5 from 83 respondents (46% of 180 NHL athletic trainers), with many positive comments on its reception.

## DISCUSSION

Once a patient has safely reached advanced medical care, definitive treatment of the vascular injuries is critical. In-hospital management has evolved over the past 20 years with the advent of endovascular treatment, which has resulted in a decrease in the 60–80% historical mortality rates. Of patients who survive transport to the hospital, carotid injuries still have at least a 30% mortality rate, additional 30% risk of stroke, and up to 85% chance of major ischemic stroke or death if the carotid is sacrificed during repair.

Hospital management of vascular neck injury is centered on resuscitation, surgical exploration, or endovascular repair. Resuscitation involves massive transfusion protocol, fluids, and vasopressors. Surgical exploration can be done with repair/reconstruction, which is preferred over ligation or sacrifice due to risk of major ischemic stroke or death if carotid scarring occurs.^24, 25^An injured jugular vein can typically be ligated or sacrificed without complication. Endovascular management options include embolization or stenting, with stenting preferred over embolization for the same reason as above.^26^

The pillars of hemostatic resuscitation include preserving tissue perfusion and clotting. The goal for mean arterial pressure is lowered to 50–60 mm Hg.^27, 28^ Additional principles include early and rapid transfusion of red blood cells and clotting factors (plasma, platelets, cryoprecipitate) in a balanced ratio, minimizing use of crystalloids, preventing hypothermia (eg, Bair Hugger warming blanket), and administering tranexamic acid.^29^ Continued digital pressure remains paramount. The wound should remain packed with plain gauze until ready for intervention. Hemostatic dressings such as Quick Clot theoretically risk an embolic event into the open carotid vessel, which could further increase stroke risk, and may not be advised.

If there are hard signs of vascular injury and the patient is hemodynamically unstable, the priority is expeditious transport to the operating room. If the patient is stable and can undergo CT angiography, that is recommended to assess the injury as some injuries are more amenable to endovascular repair. Injuries to the vertebral or subclavian arteries are difficult to access surgically and may be more suitable for endovascular treatment.^30^ Prior to transport, airway management must be considered before leaving the resuscitation bay.^29^

Medical simulation with the use of cadavers, animals, or synthetic models has been shown to be an effective tool to provide hands-on training in many different specialties.^31, 32^ In a meta-analysis by McGaghie et al, simulation-based medical education proved to be superior to traditional clinical medical education in achieving clinical skills.^33^ Unfortunately, the use of cadaveric and live-animal training models is limited by their cost, availability, and ethical and biohazard considerations. Our group has previously demonstrated the utility of selective laser-sintered, three-dimensional training models to improve skills and confidence in management of catastrophic internal carotid artery injuries in endonasal sinus surgery.^34, 35^

Maza et al concluded that surgical simulation of an arterial injury significantly decreased both time to hemostasis and blood loss. Time to hemostasis was reduced from 105.49 to 40.41 seconds (*P* < .001). The volume of blood loss was reduced from 690 to 272 mL (*P* < .001), and the confidence scores increased in 95.7% of participants, from an average of 3 to 8.^34^ Simulation also provides the ability for recurring training, feedback, reflection, and discussion. A training course consisting of education and simulation of acute vascular neck injuries may improve ability to identify type and source of hemorrhage, decrease psychomotor stress, control catastrophic bleeding, and develop effective team strategies to prevent exsanguination in the field.

In this study we aimed to address a gap in education of prehospital management of catastrophic vascular neck injuries by developing a curriculum and task simulator geared toward athletic trainers and first responders. By creating a simplified algorithm, the course could be extrapolated to target other individuals such as coaches, referees, or the public. A recent paper by Simpson et al in the *Scandinavian Journal of Trauma, Resuscitation, and Emergency Medicine* acknowledged the lack of expert agreed-upon guidance for prehospital management of penetrating neck injury after conducting a national survey of multiple professional specialists who manage PNI with the goal of aggregating statements and curating an algorithm.^36^ In our study, we introduced the curriculum and task trainer to180 NHL athletic trainers and team physicians who overwhelmingly provided positive feedback; however, due to the nature of the conference, quantitative feedback was limited. In the future, we would seek to expand this model to other sports trainers and other professionals who may be on the scene of PNI, such as police officers and firefighters. Furthermore, to increase rigor of the training we would like to implement pre- and post-model testing evaluating participants’ knowledge and comfort level in initial response to PNI.

## CONCLUSION

There remains a lack of guidance on the prehospital management of vascular neck injuries. We propose a novel algorithm for training first responders, which can be used by the public or medical professionals. For high-risk, low-frequency events, simulation of these events is a highly effective strategy to develop technical skills and communication tactics, ultimately improving outcomes. We further describe a novel, 3D-printed model that successfully facilitated a hands-on training curriculum in conjunction with the National Hockey League to provide recurring training opportunities for their athletic trainers and team physicians.

## Supplementary Information

Video 1 Simulation of a jugular vein laceration and demonstration of how to control the wound.

Video 2 Simulation of a carotid artery laceration with pulsatile, higher pressure blood flow compared to the jugular vein laceration.
